# Supplementary material for: Haplotype-aware modeling of cis-regulatory effects highlights the gaps remaining in eQTL data
Source: Nat Commun. 2024 Jan 15;15:522. doi: 10.1038/s41467-024-44710-8 (PMC10789818; doi:10.1038/s41467-024-44710-8)
Supplement: Supplementary file 1 — Supplementary Information [file 41467_2024_44710_MOESM1_ESM.pdf]

Supplementary Information

Haplotype-aware modeling of *cis*-regulatory effects highlights the gaps remaining in eQTL data

Nava Ehsan[1], Bence M. Kotis[1], Stephane E. Castel[2,3], Eric J. Song[1], Nicholas Mancuso[4], Pejman Mohammadi[1,5-7 *]

[1]Department of Integrative Structural and Computational Biology, The Scripps Research Institute, La Jolla, CA, USA

[2]Department of Systems Biology, Columbia University, New York, NY, USA

[3]New York Genome Center, New York, NY, USA

[4]Center for Genetic Epidemiology, Department of Population and Public Health Sciences, Keck School of Medicine, University of Southern California, CA, USA

[5]Center for Immunity and Immunotherapies, Seattle Children's Research Institute, Seattle, WA, USA

[6]Department of Pediatrics, University of Washington School of Medicine, Seattle, WA, USA

[7]Department of Genome Sciences, University of Washington, Seattle, WA, USA

* Corresponding author: Pejman Mohammadi (pejmanm@uw.edu)

Supplementary Figures

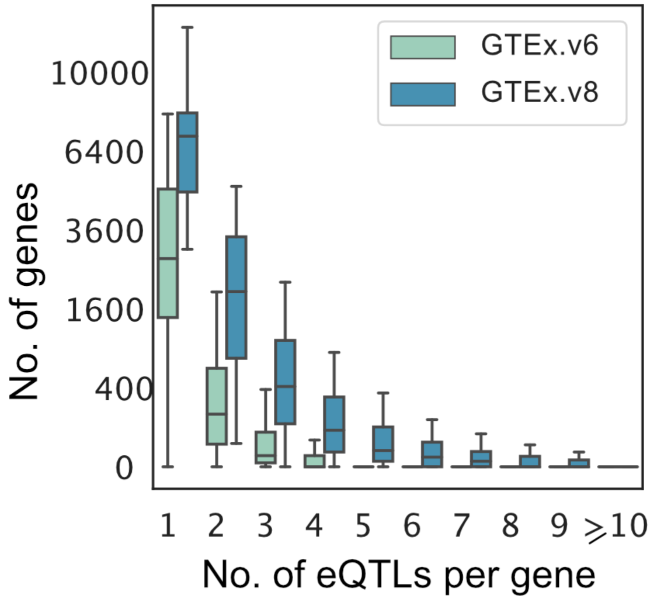

Supplementary Fig.1: Plenty of genes are associated with multiple independent regulatory variants. We compared the quantity of genes as a function of the number of associated eQTLs per gene, across tissues for GTEx v6 (185,147 genes in 43 tissues) and GTEx v8 (475,826 genes in 49 tissues) data. About 70 percent of genes have more than one eQTL in at least one tissue in GTEx v8 data. About 9.4 percent of genes in each tissue have multiple eQTLs in GTEx v6 and this increased to 25.9 percent in GTEx v8 data. There are up to 7 and 16 independent eQTLs per gene for GTEx v6 and v8, respectively. Boxplots represent first quartile, median, and third quartiles. Whiskers represent Q1-1.5* interquartile range (IQR) and, Q3 + 1.5*IQR. Outliers are hidden for ease of viewing.

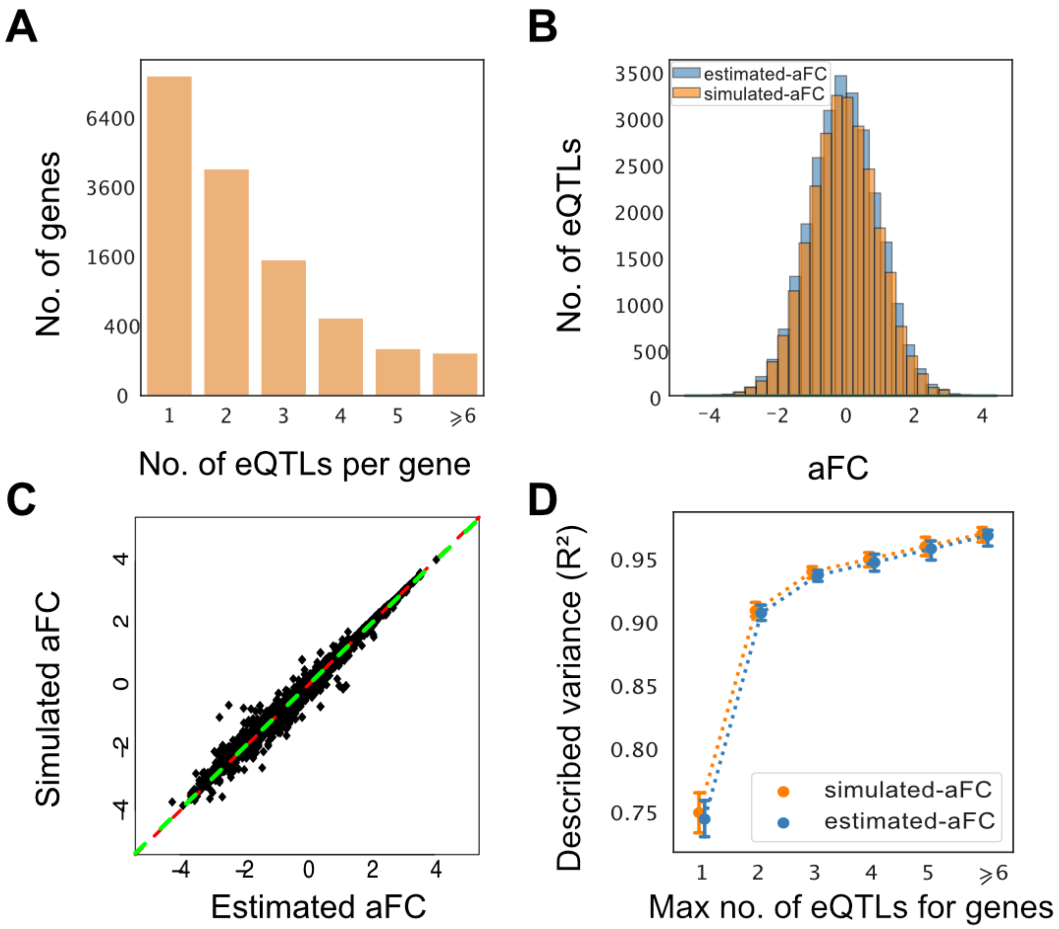

Supplementary Fig.2: Estimated effect sizes using the simulated expression data. A) Distribution of the number of associated eQTLs per gene for 15,167 genes. B-C) Our method provides highly accurate and similar estimates to simulated aFCs when all eQTL variants are included in the model. The distribution of estimated aFCs (*norm*[-0.01,σ=1.01]) is similar to the distribution of simulated aFCs (*norm*[0,σ=1]) (B). Pearson and Spearman correlation coefficients are both 0.99, and the Deming regression line shown in green is (y= 0.99x+0.00015) and the red line is (y=x) (C). D) Described variance of predicting simulated allelic imbalance with the estimated and simulated effect sizes. Error bars represent bootstrap 95% confidence intervals.

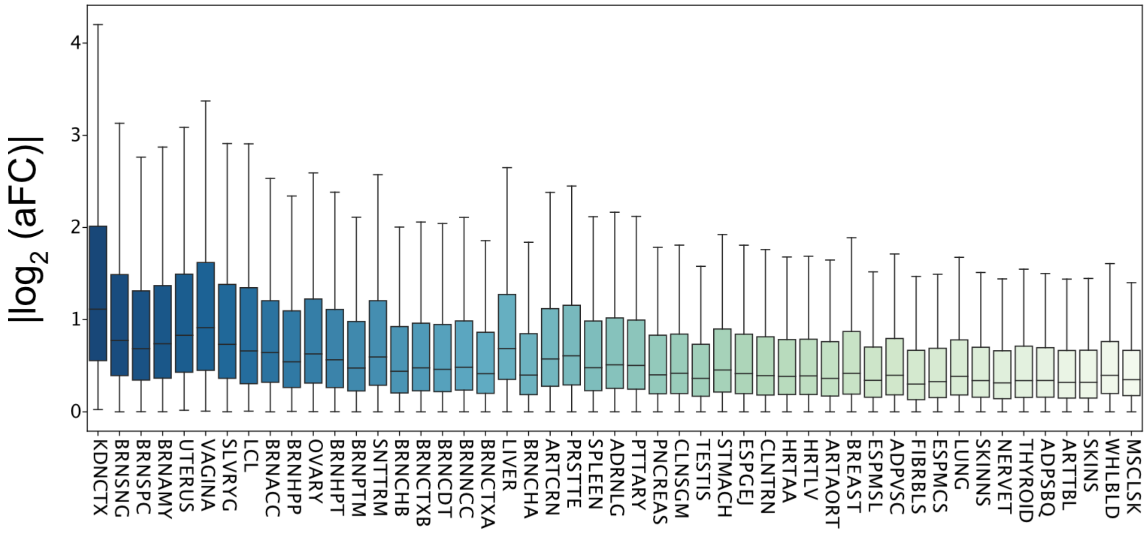

Supplementary Fig.3: Distribution of absolute aFC across tissues, sorted from smallest sample size (kidney-cortex, n = 73) to the largest (muscle-skeletal, n = 706). The distribution of aFCs for *cis*-eQTLs detected in GTEx tissues are dependent on the sample size. There is not sufficient power to detect weak eQTLs for tissues with lower sample sizes. Boxplots represent first quartile, median, and third quartiles. Whiskers represent Q1-1.5* interquartile range (IQR) and, Q3 + 1.5*IQR. Outliers are removed for ease of viewing.

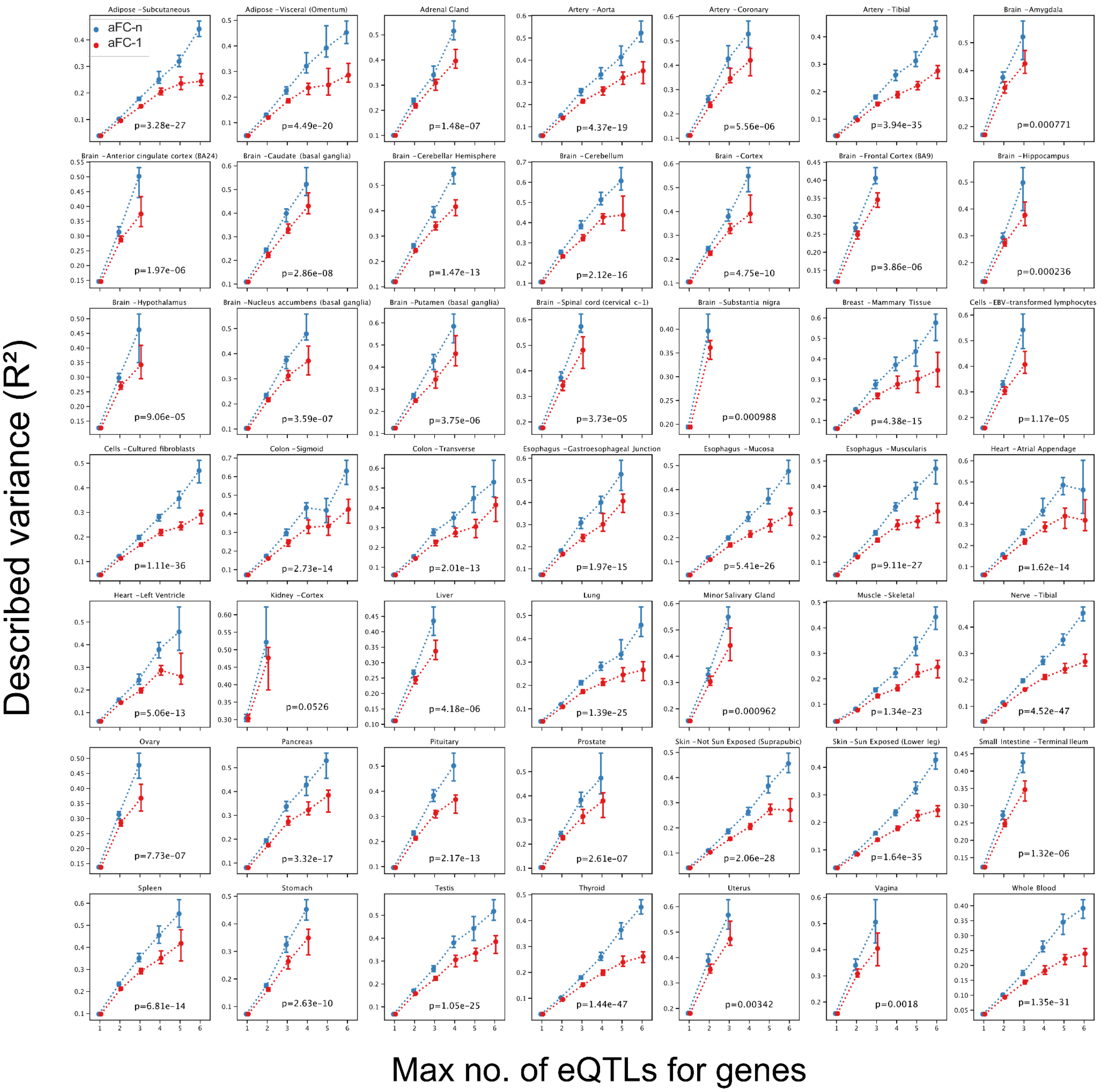

Supplementary Fig.4: The gene expression prediction accuracy gap between aFC-n and aFC-1 was widened progressively in multi-eQTL genes for 49 GTEx tissues. The genes are capped at either 6 counts or where there were more than 20 genes. The p-values represent the two-sided ranksum test for genes with more than 1 eQTL for each tissue. Error bars represent bootstrap 95% confidence intervals.

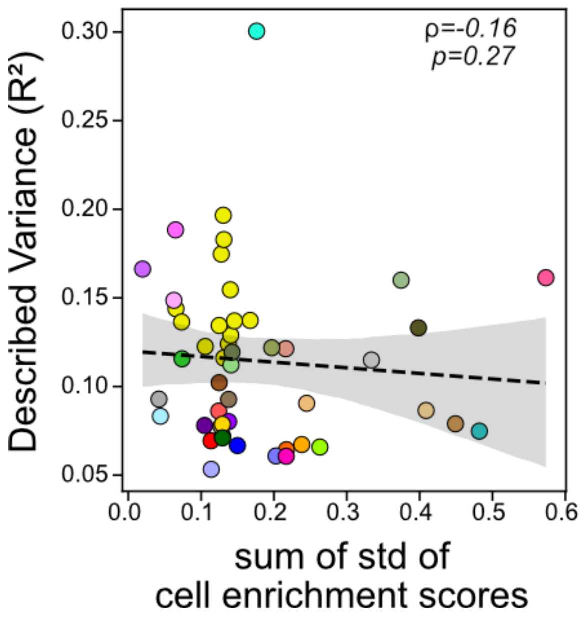

Supplementary Fig.5: We did not observe correlation between cell type heterogeneity and the median R^2 for each tissue. The x-axis is the sum of standard deviation for cell enrichment scores for 7 cell types (adipocytes, epithelial cells, hepatocytes, keratinocytes, myocytes, neurons, neutrophils) across tissue samples (17,382 samples from 49 different tissues), and the y-axis represents the median R^2 obtained from the aFC-n model (See Fig.4 for the legend of tissue colors). The dashed line represents the linear regression, ρ is the spearman correlation and, p indicates the p-value of the correlation.

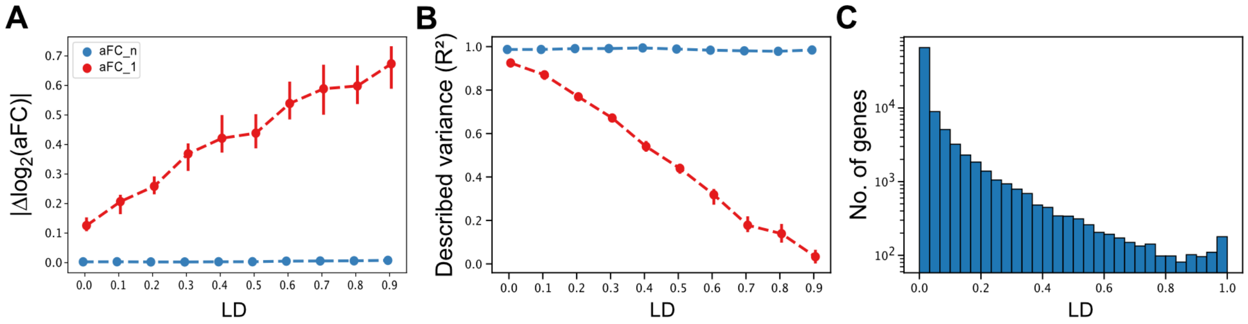

Supplementary Fig.6: The aFC-n model showed stable performance in effect size estimation and prediction accuracy on simulated data compared to aFC-1 at different levels of LD, for independent eQTL variants. A-B) The effect size estimation and prediction accuracy on simulated data. The x-axis represents different levels of LD between the eQTL variants for 2000 genes (200 genes at each level) associated with two eQTLs. The y-axis is the absolute difference between the simulated and predicted effect sizes (A) and prediction accuracy (B). Error bars represent bootstrap 95% confidence intervals. C) The distribution of LD values among the eQTL variants of 2-eQTL genes (n=98,039) in 49 GTEx tissues.

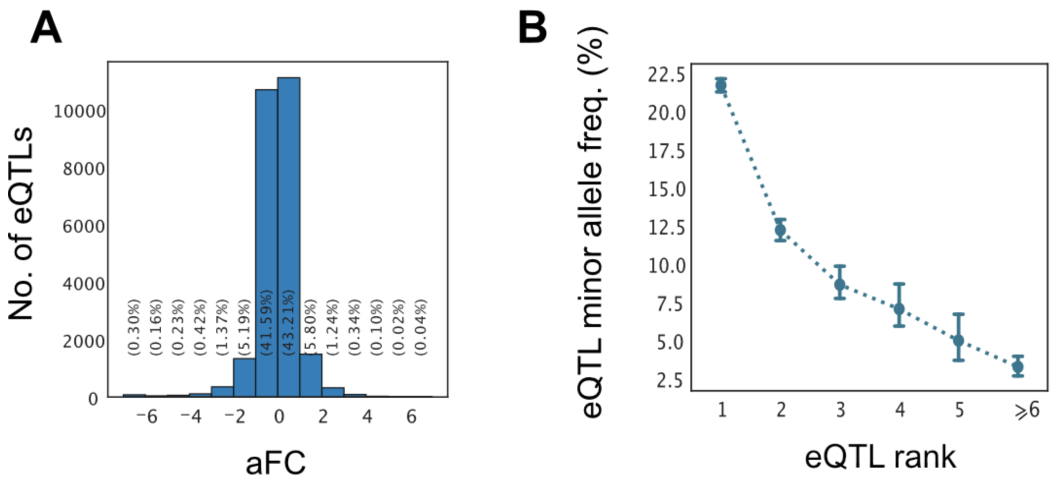

Supplementary Fig.7: Empirical properties of estimated aFCs in GTEx v8 adipose subcutaneous tissue. A) Distribution of estimated aFCs for 25,682 identified eQTLs. B) Minor allele frequency has a decreasing pattern for secondary eQTLs (41.3% of eQTLs are secondary eQTLs). The effect size distribution is affected by the power of eQTL mapping (Fig.2A-B). Error bars represent 95% bootstrap confidence intervals of the median.

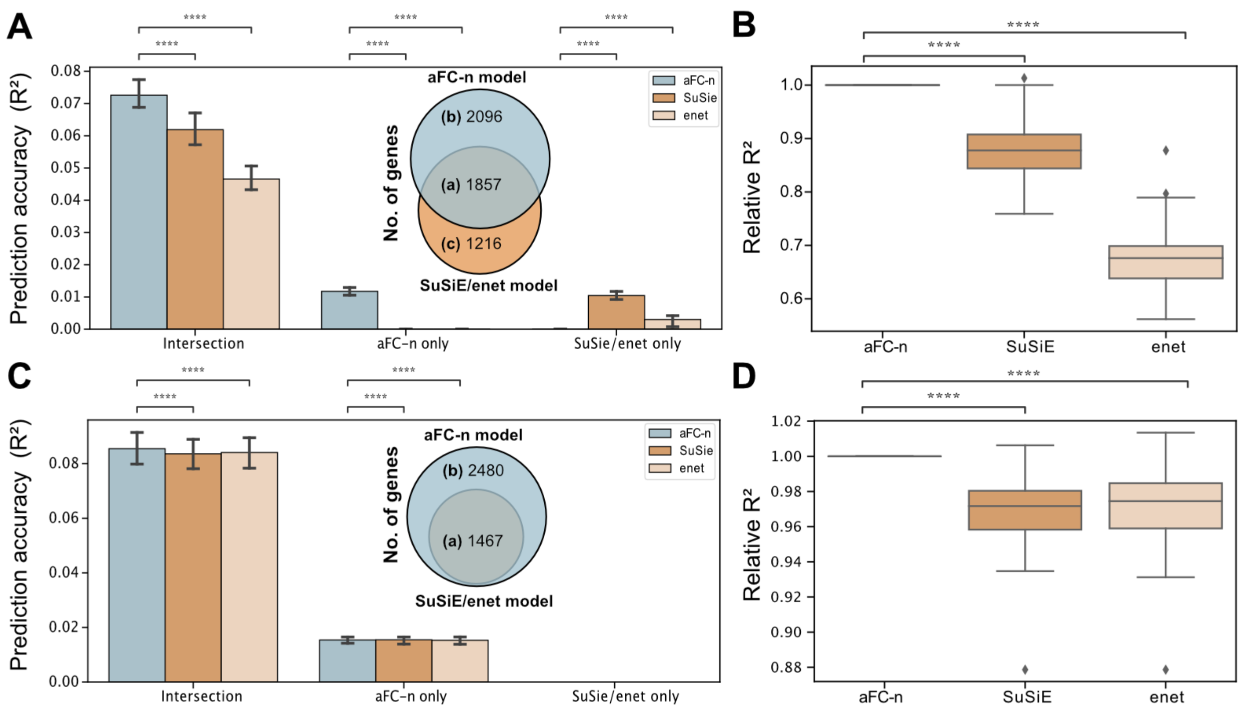

Supplementary Fig.8: Performance of aFC-n, elastic net and SuSiE applied on adjusted gene expression. We fit the models to the log-transformed and normalized gene expression data, adjusting for the top 5 genotyping PCs, sequencing protocol (PCR-based or -free), sequencing platform (Illumina HiSeq 2000 or HiSeq X) and sex. The performance is evaluated by computing the out-of-sample R^2 using newly added individuals in GTEx v8. A-B) All genetic variants in the 1Mb window around each gene are included in SuSiE and elastic net. Here we show the prediction accuracy for Adipose subcutaneous tissue (A) and the R^2 across tissues relative to the median of the aFC-n model for each tissue (B), Comparing aFC-n and SuSiE prediction models, Wilcoxon signed-rank test is significant (FDR < 0.05) for 45/47 tissues. C-D) Only conditionally independent eQTL SNPs for a gene are included in SuSiE and elastic net. (Identical set of variants used for all three methods). SuSiE and enet cannot be run with only a single SNP. Thus, we imputed the gene expression for 1-eQTL genes with ordinary least squares (OLS) method. Here we showed the prediction accuracy for 10 most sampled tissues (C) and the R^2 across tissues relative to the median of the aFC-n model for each tissue (D), Comparing aFC-n and SuSiE prediction models, Wilcoxon signed-rank test is significant (FDR < 0.05) for all tissues. The p-value annotation: ****: $p \leq 10^{-4}$. Error bars in A and C represent bootstrap 95% confidence intervals. Boxplots in B and D represent first quartile, median, and third quartiles. Whiskers represent Q1-1.5* interquartile range (IQR) and, Q3 + 1.5*IQR.

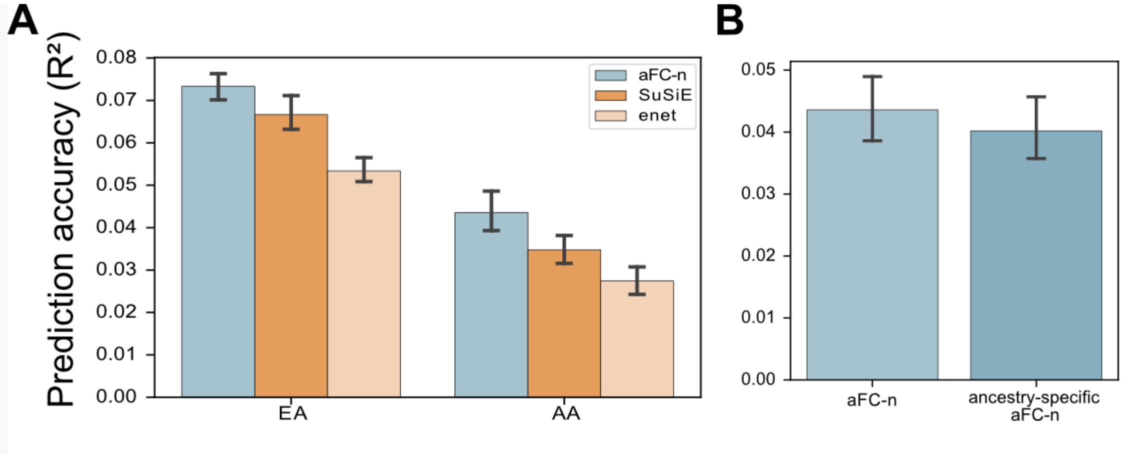

Supplementary Fig.9: The expression prediction accuracy is lower among African American (AA) compared to European American (EA) individuals. The aFCs for independent eQTLs in GTEx v6p data were derived using normalized expressions of adipose subcutaneous samples and tested on unseen GTEx v8 samples. A) Comparison of predicted gene expression with aFC-n, elastic net (enet) and SuSiE for 4,719 genes in common among all models, stratified by self-reported ancestry for the GTEx donors. B) The ancestry-specific aFC-n estimation did not improve the accuracy of the standard aFC-n model on AA expression prediction accuracy. This could be explained as the result of overfitting to the limited training data for the AA population (n=44) and also failing to reflect admixed haplotype structure in African American donors. Error bars represent bootstrap 95% confidence intervals.

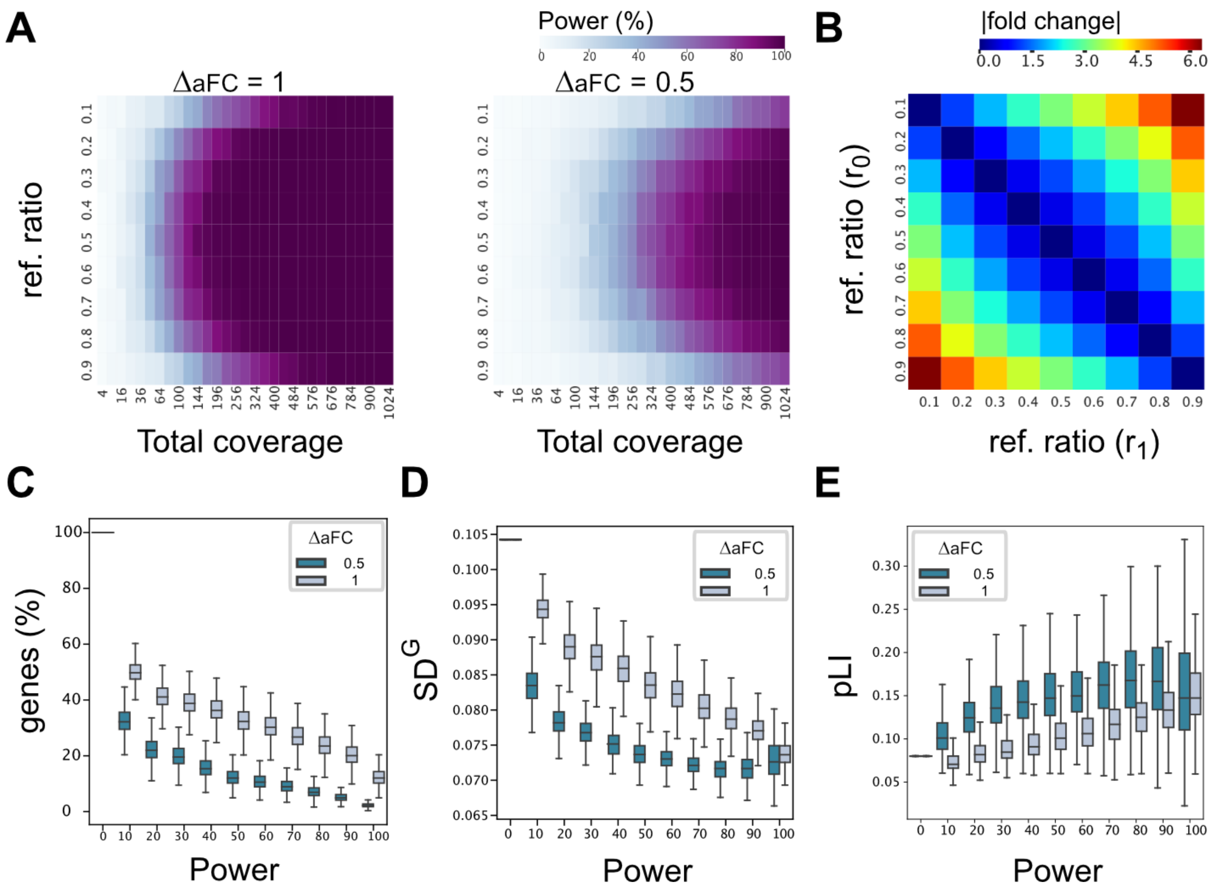

Supplementary Fig.10: Power analysis to estimate the fraction of the cases that the current eQTL data does not fully describe ASE signal. A) Power estimation based on simulation for a set of read counts and reference ratios for the specific fold changes (ΔaFC) 0.5 and 1. In low count cases there is not enough power to confirm the difference between the observed and predicted values, and improvement in power is observed by increase in the expression read counts. B) The absolute fold change between the *log*$_2$(aFC)s is illustrated as a difference between the reference ratios. The reference ratio is defined as the logistic function of the *log*(aFC) using Eq.5. C) The percentage of genes in samples (n=581) at different levels of power for adipose subcutaneous tissue. This indicates that for 6.9 and 23.5 percent (median) of genes in an individual, there is 80 percent power to detect 0.5- and 1-fold changes, respectively. The percentage of genes with an excess allelic imbalance is presented in Fig.4A. D) The SD^G is calculated for 13,965 genes in adipose subcutaneous tissue, the figure shows the median of SD^G estimates across samples as a function of power. Highly expressed genes with high statistical power tend to be also less tolerant to variation. E) Median of pLI (Probability of loss of function intolerance) across samples as a function of power for a total of 19,339 genes. Highly expressed genes with high statistical power are less tolerant to protein truncating variation. Boxplots

represent first quartile, median, and third quartiles. Whiskers represent Q1-1.5* interquartile range (IQR) and, Q3 + 1.5*IQR. Outliers are removed for ease of viewing.

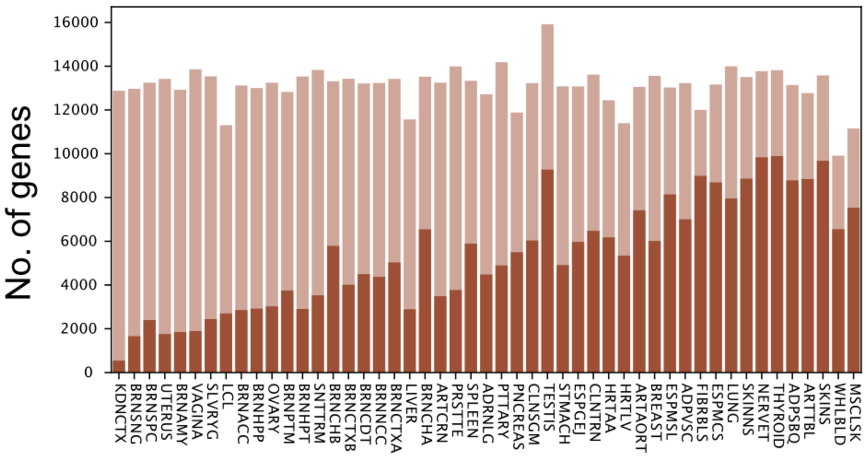

Supplementary Fig.11: The number of eGenes grows with the sample size. Amount of all protein-coding genes with median TPM >1 (shown with light bar) and the number of autosomal eGenes (shown with dark bar) per tissue (Spearman corr. = 0.92).

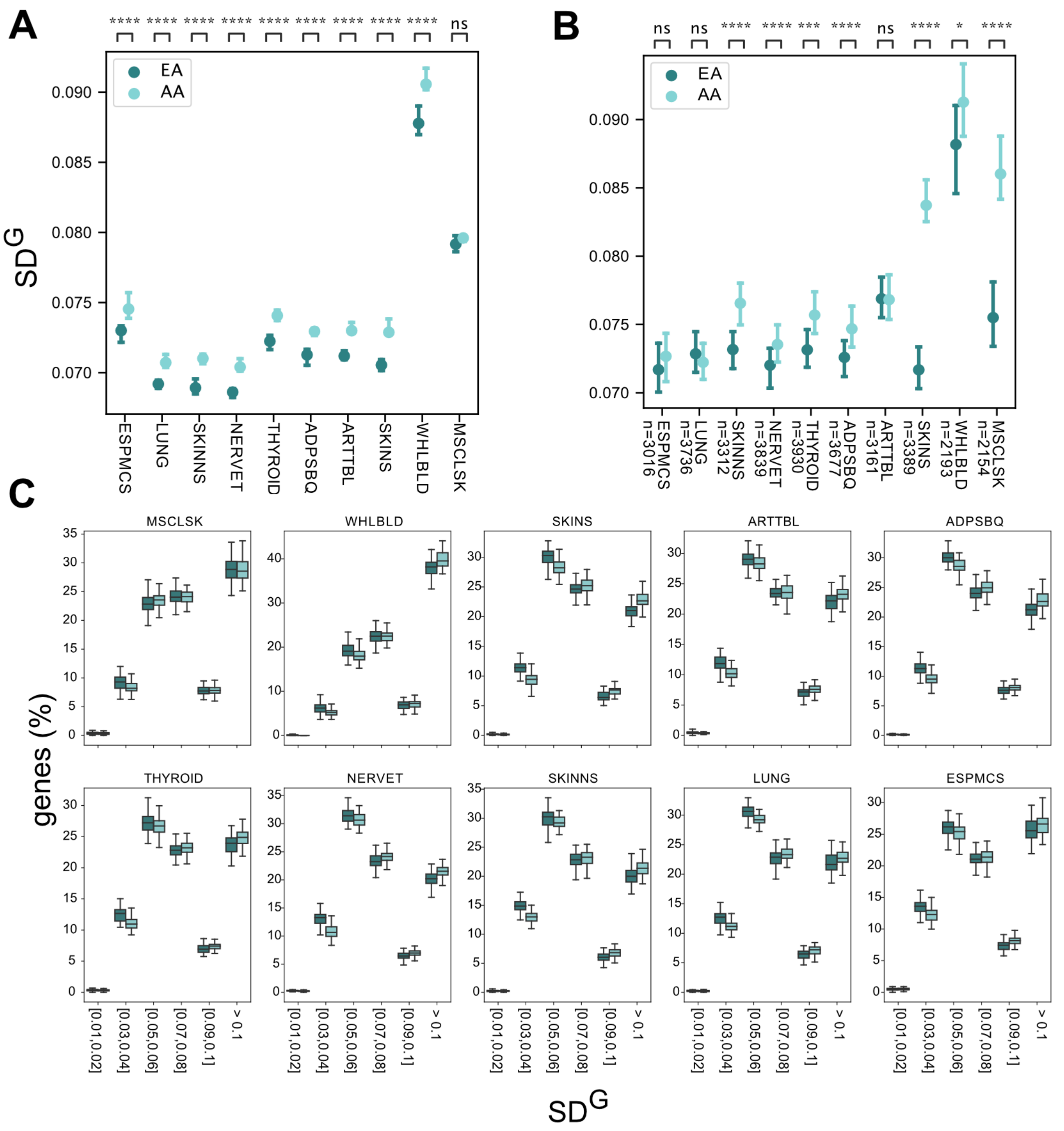

Supplementary Fig.12: SD^G estimates across African American and European American populations in top 10 sampled tissues. A-B) The genetic dosage variation is more variable in the AA population. The median of SD^{G}s across samples (A). The expression variations of the common genes in AA and EA populations present in at least one sample analyzed in (Fig.5C). The number of genes for each tissue is specified in the x-axis. The SD^{G}s are calculated over 60 random samples of each population (B). Two-sided Wilcoxon signed-rank test with Bonferroni correction p-value annotation: ns: $0.05 < p \leq 1.00$; *: $10^{-2} < p \leq 0.05$; **: $10^{-3} < p \leq 10^{-2}$; ***: $10^{-4} < p \leq 10^{-3}$; ****: $p \leq 10^{-4}$. C) Proportion of tested genes as a function of SD^G bins. The x-axis is the ancestry agnostic SD^G capped at 0.1 for ease of viewing. African Americans have a larger proportion of genes with higher variation. Error bars in A and B represent bootstrap 95%

confidence intervals. Boxplots C represent first quartile, median, and third quartiles. Whiskers represent Q1-1.5* interquartile range (IQR) and, Q3 + 1.5*IQR.

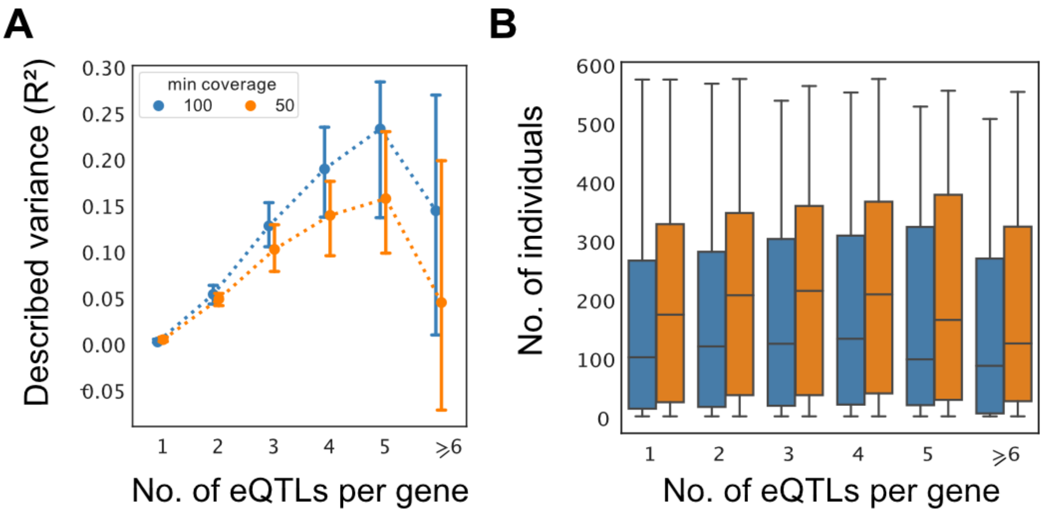

Supplementary Fig.13: Described variance and amount of ASE data available at the minimum read coverages of 100 and 50. A-B) Described variance of predicting allelic imbalance (A), and the number of individuals with ASE data (B) as a function of eQTL counts per gene subject to minimum read coverage of 100 and 50 from adipose subcutaneous tissue. Increasing minimum read coverage from 50 (11,361 genes) to 100 (9926 genes), increased the R^2 while missing 1,435 (12.6%) genes. Error bars represent 95% bootstrap confidence intervals of the median. Boxplots in B represent first quartile, median, and third quartiles. Whiskers represent Q1-1.5* interquartile range (IQR) and, Q3 + 1.5*IQR.
