# Supplementary material for: Haplotype-aware modeling of cis-regulatory effects highlights the gaps remaining in eQTL data
Source: Nat Commun. 2024 Jan 15;15:522. doi: 10.1038/s41467-024-44710-8 (PMC10789818; doi:10.1038/s41467-024-44710-8)
Supplement: Supplementary file 3 — Description of Additional Supplementary Files [file 41467_2024_44710_MOESM3_ESM.pdf]

Description of Additional Supplementary Files

File Name: Supplementary Data 1

Description: Tissue abbreviations used in the manuscript.

File Name: Supplementary Data 2

Description: The percentage of genes with an excess allelic imbalance after accounting for known eQTLs for each GTEx individual shown in Fig.4A.

File Name: Supplementary Data 3

Description: Sample size and median number of genes among samples analyzed in Fig.4B.

File Name: Supplementary Data 4

Description: The percentage of genes with an excess allelic imbalance by self-reported ancestry after accounting for known eQTLs for each GTEx individual shown in Fig.5A.

File Name: Supplementary Data 5

Description: Sample size and median number of genes among samples analyzed in Fig.5B.

File Name: Supplementary Data 6

Description: Sample size and median number of genes among samples analyzed in Fig.5C.
