# Supplementary material for: Haplotype-aware modeling of cis-regulatory effects highlights the gaps remaining in eQTL data
Source: Nat Commun. 2024 Jan 15;15:522. doi: 10.1038/s41467-024-44710-8 (PMC10789818; doi:10.1038/s41467-024-44710-8)
Supplement: Supplementary file 10 — Reporting Summary [file 41467_2024_44710_MOESM10_ESM.pdf]

nature portfolio

Corresponding author(s): Pejman Mohammadi

Last updated by author(s): Oct 12, 2023

Reporting Summary

Nature Portfolio wishes to improve the reproducibility of the work that we publish. This form provides structure for consistency and transparency in reporting. For further information on Nature Portfolio policies, see our Editorial Policies and the Editorial Policy Checklist.

Statistics

For all statistical analyses, confirm that the following items are present in the figure legend, table legend, main text, or Methods section.

n/a	Confirmed	
☐	☒	The exact sample size (*n*) for each experimental group/condition, given as a discrete number and unit of measurement
☒	☐	A statement on whether measurements were taken from distinct samples or whether the same sample was measured repeatedly
☐	☒	The statistical test(s) used AND whether they are one- or two-sided<br>*Only common tests should be described solely by name; describe more complex techniques in the Methods section.*
☐	☒	A description of all covariates tested
☐	☒	A description of any assumptions or corrections, such as tests of normality and adjustment for multiple comparisons
☐	☒	A full description of the statistical parameters including central tendency (e.g. means) or other basic estimates (e.g. regression coefficient) AND variation (e.g. standard deviation) or associated estimates of uncertainty (e.g. confidence intervals)
☐	☒	For null hypothesis testing, the test statistic (e.g. *F*, *t*, *r*) with confidence intervals, effect sizes, degrees of freedom and *P* value noted<br>*Give P values as exact values whenever suitable.*
☒	☐	For Bayesian analysis, information on the choice of priors and Markov chain Monte Carlo settings
☒	☐	For hierarchical and complex designs, identification of the appropriate level for tests and full reporting of outcomes
☐	☒	Estimates of effect sizes (e.g. Cohen's *d*, Pearson's *r*), indicating how they were calculated

Our web collection on statistics for biologists contains articles on many of the points above.

Software and code

Policy information about availability of computer code

Data collection	No custom software was used for data collection in this study.
Data analysis	We used phASER-POP package to analyze population-scale phased ASE data (https://github.com/secastel/phaser/tree/master/phaser_pop)<br>The cis-eQTL mapping for GTEx v6p samples was performed using tensorQTL package (https://github.com/broadinstitute/tensorqtl)<br>The SuSiE-R package was used to apply Sum-of-Single-Effects model.<br>Software for calculating aFC from independent eQTL data is available on ((https://github.com/PejLab/aFC-n)<br>Software for calculating predicted ASE and gene expression using allelic fold change is available on (https://github.com/PejLab/gene_expr_pred).<br>PLINK 2.0 is used to calculate linkage disequilibrium (LD) between the SNPs.<br>The other analyses were conducted in Python (version 3.7.3) and R (version 3.5.2).

For manuscripts utilizing custom algorithms or software that are central to the research but not yet described in published literature, software must be made available to editors and reviewers. We strongly encourage code deposition in a community repository (e.g. GitHub). See the Nature Portfolio guidelines for submitting code & software for further information.

Data

Policy information about availability of data

All manuscripts must include a data availability statement. This statement should provide the following information, where applicable:

- Accession codes, unique identifiers, or web links for publicly available datasets
- A description of any restrictions on data availability
- For clinical datasets or third party data, please ensure that the statement adheres to our policy

The datasets analyzed during the current study are available to authorized users via dbGaP under accession phs000424.v8.p2 (https://www.ncbi.nlm.nih.gov/projects/gap/cgi-bin/study.cgi?study_id=phs000424.v8.p2) and on the GTEx portal (http://gtexportal.org/).
The Genotype Calls (.vcf) and Allele Specific Expression (ASE) tables are protected and are not available due to data privacy laws.
The data that support the aFC estimates for all independent eQTLs in GTEx v8 are publicly available on 10.5281/zenodo.10002703.

Research involving human participants, their data, or biological material

Policy information about studies with human participants or human data. See also policy information about sex, gender (identity/presentation), and sexual orientation and race, ethnicity and racism.

Reporting on sex and gender	In this study, we did not collect data on sex or gender, nor did we conduct any analyses related to these factors. Our research primarily centers on the genetic variation within a population regardless of sex or gender distinctions.
Reporting on race, ethnicity, or other socially relevant groupings	We conducted some analyses based on self-reported ancestry for each GTEx donor.
Population characteristics	The GTEx v8 population is 32.9% Female and 67.1% male.<br>All the population characteristics of the GETx donors are available on https://gtexportal.org/home/tissueSummaryPage
Recruitment	All data used here is previously published. We did not recruit participants for this study.
Ethics oversight	Our use of GTEx data via dbgap has been approved by GTEx data access committee (Project ID 17093),

Note that full information on the approval of the study protocol must also be provided in the manuscript.

Field-specific reporting

Please select the one below that is the best fit for your research. If you are not sure, read the appropriate sections before making your selection.

☒ Life sciences ☐ Behavioural & social sciences ☐ Ecological, evolutionary & environmental sciences

For a reference copy of the document with all sections, see nature.com/documents/nr-reporting-summary-flat.pdf

Life sciences study design

All studies must disclose on these points even when the disclosure is negative.

Sample size	The analyses performed relied on existing Genotype-Tissue Expression (GTEx.v8) project data (15,201 RNAsequencing samples of 838 post-mortem donors across 49 tissue sites).<br>The sample sizes accounting for a pre-determined power for allele-specific expression analysis are reported in Supplementary Tables (S2 and S3).
Data exclusions	No data were excluded from analysis.
Replication	The effect size estimates from aFC-n were well correlated with the current effect size estimates from GTEx v8 eQTLs (GTEx Consortium 2020, Science).
Randomization	Samples were not randomized. The gene expressions were corrected for significant linear effects of confounding factors as described in the paper (see Methods).
Blinding	Blinding was not relevant to our study because we used pre-existing data from GTEx studies.

Reporting for specific materials, systems and methods

We require information from authors about some types of materials, experimental systems and methods used in many studies. Here, indicate whether each material, system or method listed is relevant to your study. If you are not sure if a list item applies to your research, read the appropriate section before selecting a response.

Materials & experimental systems

n/a	Involved in the study
☒	☐ Antibodies
☒	☐ Eukaryotic cell lines
☒	☐ Palaeontology and archaeology
☒	☐ Animals and other organisms
☒	☐ Clinical data
☒	☐ Dual use research of concern
☒	☐ Plants

Methods

n/a	Involved in the study
☒	☐ ChIP-seq
☒	☐ Flow cytometry
☒	☐ MRI-based neuroimaging
